# Pain and small fiber pathology in men with fibromyalgia syndrome

**DOI:** 10.1097/PR9.0000000000001212

**Published:** 2024-11-06

**Authors:** Betty Feulner, Franziska Gross, Dimitar Evdokimov, Rayaz A. Malik, Daniel Kampik, Nurcan Üçeyler

**Affiliations:** a Department of Neurology, University Hospital Würzburg, Würzburg, Germany; b Weill Cornell Medicine—Qatar, Education City, Qatar Foundation, Doha, Qatar; c Department of Ophthalmology, University Hospital Würzburg, Würzburg, Germany

**Keywords:** Fibromyalgia, Men, Small fiber pathology, Pain phenotype

## Abstract

**Introduction:**

Small fiber pathology may be involved in the pathophysiology of pain in women with fibromyalgia syndrome (FMS).

**Objectives:**

This prospective single-center case-control study provides detailed pain phenotyping and small fiber pathology data in a cohort of men with FMS on a morphological and functional level.

**Methods:**

Forty-two men with FMS underwent a comprehensive pain-related interview and neurological examination, a questionnaire and neurophysiological assessment, and specialized small fiber tests: skin punch biopsy, quantitative sensory testing including C-tactile afferents, and corneal confocal microscopy. Data were compared with those of healthy male controls.

**Results:**

Men with FMS reported generalized and permanent pain with additional pain attacks and a mostly pressing pain character. Intraepidermal nerve fiber density was reduced at ≥1 biopsy site in 35 of 42 (83%) men with FMS (controls: 32/65, 49%). Compared with male controls, men with FMS had elevated cold (*P* < 0.05) and warm detection thresholds (*P* < 0.001) and an increased mechanical pain threshold (*P* < 0.05) as well as an impairment of C-tactile afferents (*P* < 0.05). Corneal nerve fiber density was lower in male patients with FMS vs healthy men (*P* < 0.01). Male FMS patients with pathological skin innervation at ≥1 biopsy site compared with those with normal skin innervation had a higher clinical Widespread Pain Index (*P* < 0.05) indicating an association between the severity of cutaneous denervation and symptom load.

**Conclusion:**

We show a distinct pain phenotype and small nerve fiber dysfunction and pathology in male patients with FMS. These findings may have implications for the diagnosis and management of men with FMS.

## Abstract 2

Supplemental Digital Content is Available in the Text.

This is the first description of small fiber pathology in men with fibromyalgia syndrome.

## 1. Introduction

Fibromyalgia syndrome (FMS) is a chronic pain condition characterized by widespread musculoskeletal pain and additional symptoms such as fatigue, sleep disturbance, and cognitive impairment.^14^ The prevalence in the general population ranges from 2% to 4%, with women accounting for the majority of patients in clinical practice.^21^ However, the female:male ratio in epidemiological studies is only 1 to 2:1, and selection and confirmation bias may reinforce the underestimation of FMS in men.

Despite intensive research, the underlying pathomechanism of pain in FMS remains unclear. Whilst the primary focus of many studies was on pathology of the central nervous system as the key contributor to pain in FMS,^6,43^ peripheral nervous system involvement in a subgroup of women with FMS provided a new perspective^32,41,50^ and meanwhile small fiber pathology was confirmed in 30% to 70% of women with FMS.^20^ Small fiber pathology affects the thinly myelinated A-delta and unmyelinated C-fibers and may lead to neuropathic pain and autonomic dysfunction. These fibers have been assessed extensively in the skin^7,11,12,18,23,26,32,50^ and cornea^33,36^ of patients with FMS using specialized small fiber tests; however, the exact impact of the peripheral nervous system on pain in FMS is still a matter of debate. It was further hypothesized that there are distinct subgroups of patients with FMS based on the severity and pattern of small fiber impairment. Thus, small fiber pathology has been well characterized in several independent cohorts of women with FMS,^15,27^ but respective data from men are missing.

In a few studies, characteristics of FMS pain were investigated in both sexes, and a higher symptom load including more cases with generalized pain was reported in women than in men.^38,55^ Notably, the authors showed a greater impact of pain on male patients with FMS in terms of stronger experience and more catastrophizing thoughts about pain than in women with FMS. However, these earlier studies were overall underpowered, and the biological relevance of the presented findings remained elusive. Another study investigated sex differences in pain sensitivity and found lower pressure pain thresholds in women compared with men with FMS in a small sample.^8^ While these data suggest diverse phenotypic characteristics in patients with FMS depending on sex, studies investigating the peripheral nervous system with respect to small fiber impairment in both sexes are lacking and the presence and extent of small nerve fiber involvement together with its potential functional impact in men with FMS is unknown.

We set out to characterize pain in men with FMS and hypothesized that small fiber pathology is equally present in male patients with FMS. We have performed a prospective single-center case-control study comparing multilevel small fiber analysis of patients with healthy controls. Our data pioneer pain phenotyping and the investigation of small fiber impairment in men with FMS. This may open novel options for diagnostics and treatment of male patients with FMS.

## 2. Patients and methods

### 2.1. Recruitment of patients and controls

Between September 2014 and May 2023, 42 men with FMS (median age: 56, range: 31–75 years) were enrolled at the Department of Neurology, University Hospital Würzburg. Inclusion criteria: adult men with FMS per 1990 or 2010 American College of Rheumatology criteria.^14,53,54^ Exclusion criteria: other differential diagnoses of pain, polyneuropathy, diabetes, current inflammatory disease, cancer within the last 5 years, kidney disease, untreated thyroid dysfunction, severe psychiatric disorder, and substance dependence, pending compensation claims, and unwillingness to participate in all tests. Additional corneal exclusion: anterior eye disease, recent surgery, or hard contact lens use. Control subjects were healthy men from patients' acquaintances. Further control exclusions: polyneuropathy, glucose intolerance or diabetes, untreated thyroid dysfunction, psychiatric disorders, substance dependence, recent surgery, current inflammatory disease, and corneal issues. Skin innervation data were compared with normative values. Participants provided informed consent, and the study was approved by the Würzburg Ethics Committee (#121/14, #69/20).

### 2.2. Clinical examination, laboratory tests, and electrophysiological assessment

Patients had a detailed neurological examination and laboratory tests: blood counts, renal/liver function, thyroid hormone, C-reactive protein, vitamin B12, HbA1c, and glucose tolerance to rule out other causes. An electrophysiological assessment of the right sural and tibial nerves was done to exclude large fiber neuropathy.

We applied the German version of the Neuropathic Pain Symptom Inventory (NPSI),^4^ Graded Chronic Pain Scale (GCPS),^52^ and Pain Catastrophizing Scale (PCS).^30,45^ The current pain intensity at the time of examination was recorded using a zero (no pain) to 10 (worst pain) numeric rating scale (NRS). The Mainz Pain Staging System (MPSS or “Gerbershagen Grad”)^17^ was used to record the degree of chronification and the Fibromyalgia Impact Questionnaire (FIQ).^5^ Patients were further assessed with the “Allgemeine Depressionsskala” (ADS)^35^ and the State-Trait Anxiety Inventory (STAI-S, STAI-T).^44^ We determined the Ocular Surface Disease Index (OSDI)^39^ to screen for xerophthalmia. Patients were asked about their pain, analgesic medication, and other clinical characteristics in a separate interview.

### 2.3. Skin punch biopsy

We obtained 6-mm skin punch biopsies from the right lower leg and upper thigh of all patients for intraepidermal nerve fiber density (IENFD).^25,49^ We have used a 3-mm piece of skin punch biopsy for IENFD determination; 40-µm cryosections were immunoreacted with antibodies against the pan-axonal marker protein-gene product (PGP) 9.5 (1:1,000; Zytomed, Berlin, Germany) visualized by the fluorescent secondary antibody Cy3 (1:100; Dianova, Hamburg, Germany). Quantification of IENFD was performed blinded using a fluorescence microscope (Axiophot 2, Zeiss, Oberkochen, Germany) with an Axiocam MRm camera (Zeiss) and SPOT software (Diagnostic Instruments, Sterling Heights, MI).^25^ Data were compared with the IENFD of 55 male healthy controls for the lower leg (median age: 48 years, range: 22–76 years) and 40 healthy controls for the upper thigh (median age: 55 years, range: 23–76 years) collected in our department. The varying number of controls for distal and proximal IENFD is due to some controls consenting only to lower leg biopsies. These male controls were not part of the cohort used to establish the normative values of our laboratory.

### 2.4. Quantitative sensory testing

Quantitative sensory testing (QST) (Somedic, Hörby, Sweden) was performed at the dorsum of the right foot.^37^ We determined cold and heat detection thresholds (CDT, WDT), pain thresholds (CPT, HPT), and the ability to sense temperature changes (thermal sensory limen, TSL). We recorded paradoxical heat sensations (PHS) and assessed the mechanical detection and pain thresholds (MDT, MPT), mechanical pain sensitivity (MPS), pressure pain threshold (PPT), and vibration detection threshold (VDT). Quantitative sensory testing data were compared with those of 136 healthy male controls from our laboratory (median age: 52 years, range: 17–80 years). We used log-transformed raw values to calculate a z-score sensory profile for each QST variable (z-score = value of the patient − mean value of control subjects/standard deviation of control subjects).^28^ Negative z-scores indicated loss of function, and positive z-scores indicated gain of function.

We examined the C-tactile fiber function using a pleasant touch stimulus.^31^ A calibrated brush (Brush-05; Somedic) was applied 3 times on the dominant forearm at 3 cm/second over 12 cm. Participants rated pleasantness from −10 to +10 after each stimulation. We compared the average ratings with normative values from 31 healthy male controls (median age: 53, range: 20–79 years).

### 2.5. Corneal confocal microscopy

We performed corneal confocal microscopy (CCM) to visualize the corneal sub-basal nerve plexus (Heidelberg Retina Tomograph and Rostock Cornea Module; Heidelberg Engineering GmbH, Heidelberg, Germany).^47^ An ophthalmologist excluded pathologies, such as corneal erosion, through slit-lamp examination. One patient showed a pathology of the anterior segment of the eye such that CCM was performed in 41 men with FMS (median age: 55 years, range: 31–75 years). We anesthetized both eyes using 0.4% oxybuprocaine (Conjuncain EDO, Bausch & Lomb GmbH, Berlin, Germany) or 1% tetracaine (Minims, Bausch & Lomb GmbH) eye drops. A drop of Corneregel EDO (Bausch & Lomb GmbH) was applied to moisten each eye and the lens, and a sterile TomoCap (Heidelberg Engineering GmbH) was positioned over the lens. We obtained 3 images of the sub-basal nerve plexus per eye from each subject and used ACCMetrics software to quantify corneal nerve fiber density (NFD, no/mm^2^) and nerve fiber length (NFL, mm/mm^2^) and CCMetrics software to quantify corneal nerve branch density (NBD, no/mm^2^) (M.A. Dabbah, Imaging Science, Manchester, United Kingdom). Data were compared with those of 28 healthy male controls (median age: 51 years, range: 21–69 years).

We screened for xerophthalmia with the Schirmer test I (Haag-Streit UK, Bishop's Stortford, Hertfordshire, United Kingdom)^57^ since a more frequent occurrence of dry eye disease was observed in patients with chronic pain.^2,22,40^ Corneal sensitivity was tested using the Cochet–Bonnet aesthesiometer (Luneau Ophtalmologie, Chartres Cedex, France).^9^ A 6-mm thread length was set to stimulate the cornea, and if the subject did not perceive the stimulus, the thread was retracted in intervals of 0.5 cm to increase the pressure. Data points ≥5 cm were considered normal. The control groups each consisted of 13 healthy men for the Schirmer test (median age: 48 years, range: 24–64 years) and aesthesiometry (median age: 46 years, range: 24–64 years).

### 2.6. Statistical analysis

Statistical analysis was performed using the IBM SPSS Statistics 27 software (IBM, Ehningen, Germany). Graphs were designed with GraphPad Prism 9 (GraphPad Software, Boston, MA). For comparison of nonnormally distributed data, the nonparametric Mann–Whitney *U* test was applied, and the results are presented as median and range. For comparison of the normally distributed z-scores for QST data, a *t* test was applied, and the results are presented as mean ± standard deviation. For comparison of categorical data, we used the χ^2^ test and for correlation analysis, we performed the bivariate Spearman correlation. *P* < 0.05 was assumed significant.

## 3. Results

### 3.1. Clinical and laboratory findings

Table 1 gives baseline data characterizing our study cohort. Neurological examination, nerve conduction studies, and blood tests were normal in all patients with FMS. Overall, 10 of 42 (24%) patients had a 2-hour plasma glucose level ≥140 mg/dL on the oral glucose tolerance test indicative of impaired glucose tolerance according to the World Health Organization (WHO) and the American Diabetes Association.^1^ The results of the small fiber tests did not differ between men with FMS with normal and impaired glucose tolerance (see Supplementary Table 1, http://links.lww.com/PR9/A263). The median body mass index (BMI) was higher in the group of men with FMS compared with male healthy controls (FMS: 29.1 kg/m^2^, range: 21.3–45.6, controls: 25.2 kg/m^2^, range: 18.7–32.7; *P* < 0.01).

**Table 1:** Clinical data and pain characteristics of the study cohort.

	All patients with FMS (n = 42)	Normal IENFD (n = 7)	Generalized reduction of IENFD (n = 22)
Age (y)	56 (31–75)	51 (31–64)	55 (33–75)
BMI (kg/m^2^)	29.1 (21.3–45.6)	28.7 (21.6–35.5)	29.6 (23.0–39.9)
FMS criteria fulfilled			
ACR 1990	30/42 (71%)	6/7 (86%)	16/22 (73%)
ACR 2010	41/42 (98%)	7/7 (100%)	22/22 (100%)
German S3 guideline	42/42 (100%)	7/7 (100%)	22/22 (100%)
Time since diagnosis (y)	5 (1–40)	2 (1–14)	5 (1–40)
Pain duration (y)	10 (2–60)	6 (2–35)	20 (3–50)
Current pain intensity on NRS	5 (1–8)	4 (2–7)	5 (2–7)
Life event	19/42 (45%)	4/7 (57%)	10/22 (46%)
Employment status			
Regularly working	19/42 (45%)	5/7 (71%)	10/22 (46%)
Retired due to pain	8/42 (19%)	1/7 (14%)	5/22 (23%)
Sick leave due to pain	8/42 (19%)	1/7 (14%)	3/22 (14%)
Pain distribution			
Generalized	31/42 (74%)	3/7 (43%)	18/22 (82%)
Proximal	8/42 (19%)	4/7 (57%)	2/22 (9%)
Distal	3/42 (7%)	0/7 (0%)	2/22 (9%)
Pain symmetry			
Bilateral pain	37/42 (88%)	6/7 (86%)	19/22 (86%)
Unilateral pain	5/42 (12%)	1/7 (14%)	3/22 (14%)
Pain dynamics			
Permanent pain with attacks	32/42 (76%)	6/7 (86%)	17/22 (77%)
Permanent pain	7/42 (17%)	1/7 (14%)	2/22 (9%)
Pain attacks	3/42 (7%)	0/7 (0%)	3/22 (14%)
Top 3 pain characters			
Pressing	26/42 (62%)	5/7 (71%)	12/22 (55%)
Like muscle soreness	21/42 (50%)	3/7 (43%)	13/22 (59%)
Tearing	18/42 (43%)	2/7 (29%)	10/22 (46%)
Analgesic medication			
None	6/42 (14%)	1/7 (14%)	3/22 (14%)
Monotherapy	23/42 (55%)	5/7 (71%)	11/22 (50%)
Combination of ≥2	13/42 (31%)	1/7 (14%)	8/22 (36%)
Psychiatric or psychological treatment			
None	20/42 (48%)	5/7 (71%)	9/22 (41%)
Currently ongoing	7/42 (17%)	2/7 (29%)	4/22 (18%)
In the past	12/42 (29%)	0/7 (0%)	7/22 (32%)

Data are given as median with range in brackets.

ACR, American College of Rheumatology; BMI, body mass index; FMS, fibromyalgia syndrome; IENFD, intraepidermal nerve fiber density; NRS, numeric rating scale.

### 3.2. Pain in men with fibromyalgia syndrome is generalized and interferes with daily life and working capacity

Data on pain, analgesic medication, and other clinical characteristics of the patient cohort with FMS is provided in Table 1. Men with FMS mostly described bilateral (37/42, 88%) and permanent pain with additional pain attacks (32/42, 76%) over the whole body (31/42, 74%). Pain character was most frequently described as pressing (26/42, 62%), like muscle soreness (21/42, 50%) or tearing (18/42, 43%), whilst some patients reported a burning (15/42, 36%) or stabbing pain (15/42, 36%). Overall, 36 of 42 (86%) patients were taking analgesic medication mostly as monotherapy (23/42, 55%); 7 of 42 (17%) patients with FMS were currently undergoing psychiatric or psychological treatment, and 12 of 42 (29%) patients had received psychotherapy in the past. In addition, 19 of 42 (45%) patients reported a life event defined as a “very positive” or “very negative” experience that the patient subjectively associated with the first occurrence of FMS symptoms; 23 of 42 (55%) men with FMS were not pursuing permanent employment, 8 of 42 (19%) patients were currently on sick leave due to pain, and 8 of 42 (19%) patients were retired due to chronic pain.

Table 2 provides the results of the questionnaire evaluation of the study cohort. The NPSI revealed a higher sum score and discriminative score in the patient cohort compared with healthy control subjects indicating a distinct neuropathic pain component in FMS pain (*P* < 0.001 each). The graded Chronic Pain Scale and Pain Catastrophizing Scale showed a greater impairment due to chronic pain and more pronounced pain catastrophizing in the male FMS cohort in comparison with male healthy controls (*P* < 0.001 each). Men with FMS had a high degree of pain chronification according to the MPSS and showed more depressive symptoms than healthy men (*P* < 0.001). By contrast, male patients with FMS did not have a higher level of anxiety compared with male controls as assessed with STAI (*P* > 0.05).

**Table 2:** Questionnaire results of the study cohort.

	FMS (n = 42)	Healthy controls (n = 17)
NPSI sum score	3.7 (0–8)***	0 (0–3)
NPSI discriminative score	65.7 (42.0–84.3)***	42.4 (37.5–68.7)
GCPS		
Pain intensity	63 (37–93)***	0 (0–33)
Disability due to pain	52 (0–83)***	0 (0–10)
PCS sum score	21 (2–42)***	0 (0–23)
ADS sum score	20 (7–48)***	8 (0–17)
FIQ sum score	45 (24–67)***	5 (0–26)
STAI-S	41 (30–73)	41 (0–59)
STAI-T	43 (31–73)	40 (0–54)
MPSS Gerbershagen grad	3 (2–3)	/
OSDI	13 (0–60)**	2 (0–29)

Data are given as median with range in brackets. **P < 0.01, ***P < 0.001.

ADS, Allgemeine Depressionsskala; FMS, fibromyalgia syndrome; FIQ, Fibromyalgia Impact Questionnaire; GCPS, Graded Chronic Pain Scale; IENFD, intraepidermal nerve fiber density; MPSS, Mainz Pain Staging System; NPSI, Neuropathic Pain Symptom Inventory; OSDI, Ocular Surface Disease Index; PCS, Pain Catastrophizing Scale; STAI-S, State-Trait Anxiety Inventory—State; STAI-T, State-Trait Anxiety Inventory—Trait.

### 3.3. Skin innervation is reduced in subgroups of men with fibromyalgia syndrome following distinct patterns

**Figure 1.**
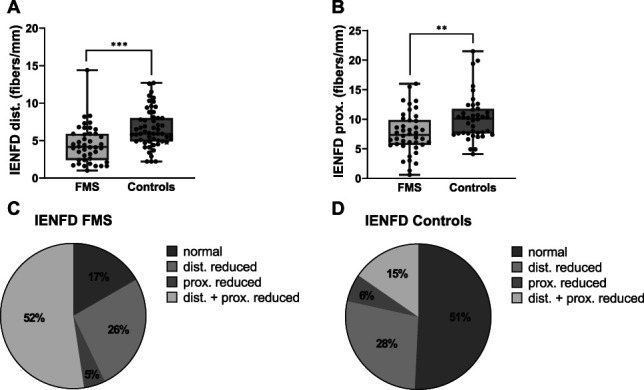
Skin innervation in men with FMS compared with healthy male controls. (A) Distal and (B) proximal IENFD was reduced in male patients with FMS compared with 55 male healthy controls for the lower leg and 40 controls for the upper thigh. We categorized 42 men with FMS and 65 male control subjects into 4 subgroups based on the skin innervation pattern. (C) IENFD was reduced at ≥1 biopsy site in 83% of male patients with FMS and (D) 49% of healthy controls. **P < 0.01, ***P < 0.001. FMS, fibromyalgia syndrome; IENFD, intraepidermal nerve fiber density.

Distal IENFD was reduced in men with FMS compared with healthy controls (FMS: 4.2 fibers/mm, range 1.0–14.4, healthy controls: 5.9 fibers/mm, range 2.2–12.7; *P* < 0.001; Fig. 1A). Proximal IENFD in men with FMS was also lower than in healthy controls (FMS: 7.4 fibers/mm, range 0.6–16.0, healthy controls: 10.1 fibers/mm, range 4.1–21.5; *P* < 0.01; Fig. 1B). There was no relationship between IENFD at the lower leg or upper thigh and age in patients with FMS or healthy controls, and it did not correlate with BMI or duration of pain (see Supplementary Table 2, http://links.lww.com/PR9/A263). Male patients with FMS with an ADS score ≥16, indicative of clinically relevant depressive symptoms, showed no differences in distal or proximal IENFD compared with patients with a score <16 (ADS ≥16: proximal IENFD 6.8 fibers/mm, distal IENFD 3.8 fibers/mm, ADS <16: proximal IENFD 7.9 fibers/mm, IENFD distal 4.6 fibers/mm; *P* > 0.05).

Based on our laboratory normative values (distal IENFD: 9 fibers/mm ± 3 fibers/mm; proximal IENFD: 12 fibers/mm ± 4 fibers/mm, see Supplementary Table 2, http://links.lww.com/PR9/A263), we defined a nerve fiber density <6 fibers/mm as pathological at the lower leg and <8 fibers/mm as pathological at the upper thigh. Based on these cut-off values, we divided the cohort of men with FMS into 4 distinct subgroups (Fig. 1C and D), namely patients with generalized reduction of IENFD (FMS: 22/42, 52%, controls: 10/65, 15%), only proximally reduced IENFD (FMS: 2/42, 5%, controls: 4/65, 6%), only distally reduced IENFD (FMS: 11/42, 26%, controls: 18/65, 28%), and normal skin innervation (FMS: 7/42, 17%, controls: 33/65, 51%).

### 3.4. Men with fibromyalgia syndrome have impaired perception of thermal and mechanical stimuli

**Figure 2.**
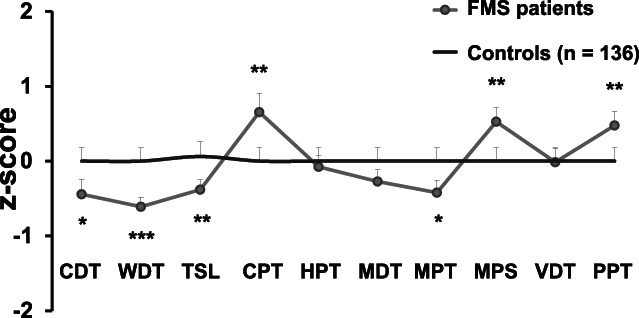
Sensory profile of men with FMS compared with male healthy controls measured with QST at the right dorsal foot. Male patients with FMS (n = 42) had elevated detection thresholds for cold (CDT) and warm (WDT) and an impaired ability to perceive temperature changes (TSL) in comparison with 136 male healthy controls. They also showed hypersensitivity to painful cold (CPT) and had an elevated mechanical pain threshold (MPT), while mechanical pain sensitivity (MDS) and pressure pain threshold (PPT) were decreased. *P < 0.05, **P < 0.01, ***P < 0.001. FMS, fibromyalgia syndrome; HPT, heat pain threshold; MDT, mechanical detection threshold; QST, quantitative sensory testing; VDT, vibration detection threshold.

Men with FMS had elevated detection thresholds for cold (CDT; *P* < 0.05) and warm (WDT; *P* < 0.001) and showed hypersensitivity to painful cold (CPT; *P* < 0.01) in QST compared with healthy men. The ability to perceive temperature changes was also impaired in the patient cohort compared with controls (TSL; *P* < 0.01). Male patients with FMS had elevated MPT (*P* < 0.05) and showed hypersensitivity to mechanical stimulation (MPS; *P* < 0.01) and blunt pressure compared with controls (PPT; *P* < 0.01; Fig. 2).

Overall, 6 of 42 (14%) men with FMS perceived the mechanical pleasant touch stimulation of C-tactile fibers on the dorsal forearm as neutral to unpleasant (ie, a score of 0 to −10). By contrast, 0 of 31 (0%) male healthy controls graded the brush stimulus between 0 and -10 (Chi^2^: *P* < 0.05).

### 3.5. Corneal innervation is reduced in men with fibromyalgia syndrome

**Figure 3.**
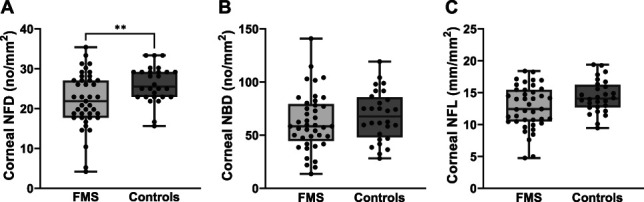
Corneal innervation in men with FMS compared with healthy male controls. (A) Corneal sub-basal NFD was reduced in male patients with FMS, while (B) NBD and (C) NFL revealed no intergroup difference between men with FMS (n = 41) and male healthy controls (n = 28). **P < 0.01. CCM, corneal confocal microscopy; FMS, fibromyalgia syndrome; NBD, nerve branch density; NFD, nerve fiber density; NFL, nerve fiber length.

The corneal sub-basal NFD was lower in male patients with FMS compared with healthy controls (*P* < 0.01) but with no difference in corneal NBD and NFL (Fig. 3). There was no correlation between the age, duration of pain, and corneal innervation in the patient cohort (see Supplementary Table 3, http://links.lww.com/PR9/A263). When comparing with published CCM normative reference values,^46^ 3 of 41 (7%) men with FMS and 1 of 28 (4%) healthy men showed a pathologically reduced NFD, 1 of 41 (2%) men with FMS and none of the controls had a pathologically reduced NBD, and 21 of 41 (51%) men with FMS and 10 of 28 (36%) healthy men had a pathological reduced NFL. Application of these reference values did not reveal intergroup differences.

Corneal sensitivity was normal in all but one man with FMS and one healthy control each; hence, we did not find a difference between groups. The Schirmer test I was positive for xerophthalmia in 11 of 41 (27%) male patients with FMS and 3 of 13 (23%) male control subjects (*P* > 0.05). No correlation was found between xerophthalmia and corneal innervation in the patient or control cohort.

### 3.6. Male fibromyalgia syndrome patients with cutaneous denervation report more widespread pain

**Figure 4.**
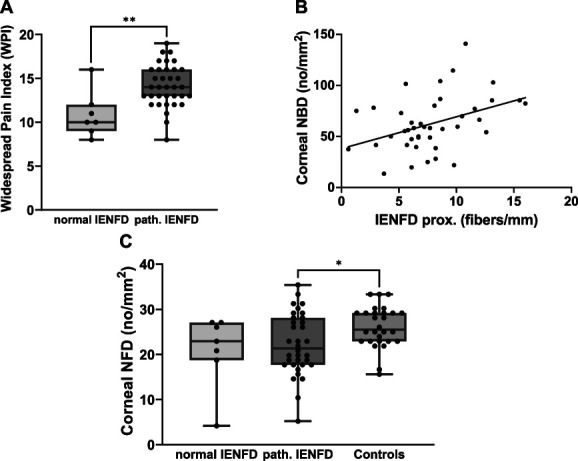
Association of the skin innervation pattern with the clinical phenotype and correlation of cutaneous and corneal denervation in men with FMS. (A) In comparison with male FMS patients with a normal IENFD (n = 7), men with FMS with pathological reduction in skin innervation at ≥1 biopsy site (n = 35) had a higher WPI. (B) The corneal sub-basal NBD correlated with the IENFD at the upper thigh in the cohort of men with FMS (r = 0.402, *P* < 0.01). (C) The corneal sub-basal NFD was reduced in the group of men with FMS with pathological reduction in skin innervation at ≥1 biopsy site (n = 35) in comparison with male healthy controls (n = 28), whereas this difference was not seen in patients with a normal cutaneous innervation (n = 7). *P < 0.05, **P < 0.01. FMS, fibromyalgia syndrome; IENFD, intraepidermal nerve fiber density; NBD, nerve branch density; NFD, nerve fiber density; WPI, widespread pain index.

We analysed whether the skin innervation pattern was associated with a distinct clinical phenotype in men with FMS. Supplementary Table 4, http://links.lww.com/PR9/A263, provides the data on questionnaire results and small fiber tests in the FMS subgroups based on their skin innervation pattern. Patients with FMS and pathological skin innervation (only distal, only proximal, and generalized reduction of IENFD) had a higher clinical Widespread Pain Index (WPI)^53^ compared with patients with normal skin innervation (*P* < 0.01; Fig. 4A). Comparing the individual subgroups, this was also true for patients with generalized (*P* < 0.05) as well as mere distal reduction of IENFD (*P* < 0.01). Both of these patient subgroups reached a higher WPI than patients with a normal IENFD. Male FMS patients with generalized skin denervation had a higher PCS score than patients with only proximally reduced IENFD (*P* < 0.05).

Subgroup analysis of the pleasant touch data revealed no differences in the patient cohort. Quantitative sensory testing sensory profiles did not differ between male FMS patients with pathological and normal skin innervation. However, men with FMS with a mere proximal reduction in IENFD had a lower mechanical pain threshold (MPT) compared with patients with a distal reduction in IENFD (*P* < 0.05), a generalized reduction in IENFD (*P* < 0.05), and patients with normal skin innervation (*P* < 0.01). Male patients with only a proximal reduction in IENFD had a lower warm detection threshold (WDT) in comparison with patients with a distal reduction in IENFD (*P* < 0.05) and a generalized reduction in IENFD (*P* < 0.05).

### 3.7. Corneal innervation correlates with skin innervation in men with fibromyalgia syndrome

Comparing corneal NFD, NBD, and NFL among the 4 subgroups of skin innervation patterns in men with FMS (see Supplementary Table 4, http://links.lww.com/PR9/A263), male FMS patients with a generalized reduction of IENFD had a lower corneal NBD compared with patients with a mere distal denervation (*P* < 0.05). Corneal NBD correlated with proximal IENFD (r = 0.402, *P* < 0.01; Fig. 4B), but there was no correlation with corneal NFD or NFL. Looking at the larger cohort of male patients with a pathologically reduced IENFD (only distal, only proximal, and generalized reduction of IENFD), men with FMS had a lower corneal NFD in comparison with the group of male healthy controls, whereas this difference was absent in patients with normal skin innervation (*P* < 0.05; Fig. 4C).

## 4. Discussion

We have undertaken detailed clinical pain phenotyping and assessment of small nerve fiber dysfunction and skin and corneal nerve innervation to identify unique pathology in men with FMS. We report distinct characteristics of pain and small fiber pathology in the majority of male patients with FMS as reflected by cutaneous denervation as well as an impairment of small nerve fiber function. We further found patterns of skin denervation showing defined characteristics in the remaining small fiber tests.

Our data reveal that men with FMS primarily report generalized and permanent pain with additional pain attacks of mostly a pressing character. These data are in line with the pain phenotype of women with FMS as previously reported,^15^ but in contrast with earlier studies, which identified a mostly stabbing and localized rather than widespread pain in men with FMS.^38^ The discrepancy in findings may be due to the overall small sample sizes. We also found evidence for a neuropathic pain component in FMS pain and a high degree of pain chronification that has a major impact on daily life as reflected by high FIQ scores and on careers with 55% of patients not working regularly. A high proportion of men with FMS displayed depressive symptoms and more catastrophic thoughts about pain; however, nearly 50% never received psychotherapy. Earlier studies^38,55^ also showed high FIQ scores in male patients with FMS indicating a great impact on pain, which is in line with our results. Furthermore, the median current pain intensity was 5 of 10 NRS and 86% of men with FMS were on analgesic medication, whilst in a previous study only 76% of women with FMS with a comparable median pain intensity were taking analgesics.^15^

Impaired glucose tolerance occurred in almost a quarter of men with FMS, comparable to a previous study in female patients with FMS,^15^ but was not related to the extent of small fiber dysfunction or damage. There is controversy regarding the effect of glucose dysmetabolism on small nerve fibers^34,42^ and symptom severity^16,48,56^ in FMS. However, there are data showing a relationship between glucose tolerance and alterations in IENFD as well as corneal nerve fiber morphology.^3^ While in our study there was no correlation between BMI and skin innervation in male patients with FMS, a higher BMI impacts glucose metabolism and symptom severity in FMS,^10,51,56^ suggesting that weight loss could improve glucose metabolism and reduce pain and additional symptoms in patients with FMS.^10^

In previous studies, FMS subgroups have been defined based on the extent of alterations in small fibers in skin and cornea^33^; however, hardly any male patients were included. We have found distinct skin innervation patterns and an interrelation between corneal and cutaneous innervation independent of age or pain duration in men with FMS. Comparing skin innervation patterns in our male patient cohort with those of women with FMS investigated in our previous study,^15^ it is striking that >50% had a generalized reduction in skin innervation, while this occurred in only 15% of women. Furthermore, mere proximal denervation was hardly found in male patients with FMS, whilst this was frequent in women with FMS, indicating that these sex differences in skin innervation warrant further investigation.

In line with previous studies mainly performed in women with FMS,^12,15,26,33,50^ we found evidence for impaired small nerve fiber function in male patients with FMS. This was reflected by elevated thermal detection thresholds, increased mechanical pain thresholds, and alterations in the pleasant touch test. Previous data on sensory profiles in FMS are quite divergent,^12,15,26,27,33,50^ which may be due to diversity in the normative reference values and subjectivity of this standardized, but psychophysical method.

With regard to corneal small fiber pathology, there was a reduction in ≥1 of the CCM parameters in 51% of men with FMS referred to normative reference values, indicating widespread small nerve fiber damage. This confirms the findings of an earlier study using CCM to construct phenotypes in a cohort of predominantly female patients with FMS,^33^ where the authors showed a loss of corneal nerve fibers but no correlation between abnormal corneal morphology and QST, WPI, and disease duration. Another CCM study in women with FMS also reported no correlation between corneal nerve fiber parameters and the results of FMS-specific questionnaires.^36^ In this study, we show that WPI differentiated best between men with FMS with a pathological compared with normal skin innervation. The lack of correlation between quantitative small fiber reduction and dysfunction in the remaining small fiber tests, namely QST and pleasant touch test, as well as questionnaire data was also reported.^15,27,33^ The exact role of small fiber pathology regarding somatosensory function and symptoms of patients with FMS thus remains unclear.

A limitation of our study is the rather small sample of men with FMS. Nevertheless, to date, this is the largest cohort of male patients with FMS systematically examined with regard to clinical characteristics and small nerve fiber impairment. Furthermore, several healthy men in our control cohort had a reduced IENFD as already shown in previous studies^13,19^ although all participants were screened negative for known causes of small fiber pathology. Our normative values for IENFD are already based merely on one standard deviation from the mean, considering that published studies addressing normative values for IENFD reported similar or even higher cut-off values.^19,24,29^ Our study is the first to investigate a cohort of men with FMS for pain characterization and small fiber assessment on a morphological and functional level. This has enabled us to establish a distinct pain phenotype in men with FMS which is similar to that in women, while we show skin innervation patterns in male patients with FMS which differ from female patients with FMS. Furthermore, we show that the severity of skin denervation correlates with pain severity and corneal denervation. Our findings may improve the understanding of the pathophysiology, diagnosis, and clinical management of FMS in men. In addition, identifying pathological cutaneous and corneal innervation as measurable and objective findings may help patients to better accept and cope with this chronic pain condition.

## Disclosures

The authors have no conflict of interest to declare.

## Appendix A. Supplemental digital content

Supplemental digital content associated with this article can be found online at http://links.lww.com/PR9/A263.

## Supplementary Material

SUPPLEMENTARY MATERIAL
