# Supplementary material for: Pain and small fiber pathology in men with fibromyalgia syndrome
Source: Pain Rep. 2024 Nov 6;9(6):e1212. doi: 10.1097/PR9.0000000000001212 (PMC11543218; doi:10.1097/PR9.0000000000001212)
Supplement: SUPPLEMENTARY MATERIAL [file painreports-9-e1212-s001.pdf]

Supplementary Table 1. Clinical data, results of questionnaire assessment, and small fiber tests in men with FMS with normal and impaired glucose tolerance**.**

	**Normal glucose tolerance (n = 32)**	**Impaired glucose tolerance (n = 10)**
Age (years)	55 (33 – 75)	61 (31 – 74)
BMI (kg/m^2)	28.9 (21.3 – 40.6)	30.1 (23.0 – 45.6)
Pain duration (years)	10 (2 – 60)	20 (4 – 50)
Current pain intensity on NRS	5 (1 – 8)	5 (2 – 8)
NPSI sum score<br>NPSI discriminative score	3.7 (0 – 7.4)<br>66.7 (42.4 – 84.3)	2.6 (0 – 8.0)<br>54.5 (42.0 – 79.6)
GCPS<br>– Pain intensity<br>– Disability due to pain	 65 (37 – 93)<br>49 (0 – 83)	 62 (42 – 83)<br>62 (8 – 80)
PCS sum sore	20 (2 – 41)	24 (2 – 42)
ADS sum sore	18 (7 – 39)	24 (8 – 48)
FIQ sum score	45 (27 – 67)	48 (24 – 66)
STAI-S<br>STAI-T	43 (32 – 73)<br>43 (31 – 73)	38 (30 – 48)*<br>42 (34 – 55)
MPSS Gerbershagen Grad	3 (2 – 3)	3 (3 – 3)
WPI<br>SSS	13.5 (8 – 19)<br>8 (3 – 11)	13.5 (8 – 18)<br>6.5 (5 – 12)
**Small fiber tests**		
IENFD (fibers/mm)<br>– distal	 4.3 (1.0 – 8.3)	 4.2 (1.6 – 14.4)

– proximal	7.6 (0.6 – 15.5)	6.4 (2.5 – 16.0)
IENFD subgroups		
– Normal IENFD	6/32 (19%)	1/10 (10%)
– Proximally reduced	1/32 (3%)	1/10 (10%)
– Distally reduced	9/32 (28%)	2/10 (20%)
– Generalized reduction	16/32 (50%)	6/10 (60%)
CCM		
– NFD (no/mm^2)	22.4 (4.2 – 35.4)	19.8 (5.2 – 33.3)
– NBD (no/mm^2)	56.8 (19.8 – 114.6)	63.5 (13.5 – 140.8)
– NFL (mm/mm^2)	12.8 (5.0 – 18.4)	12.4 (4.8 – 18.3)
QST		
– CDT	-0.29 (-2.26 – 1.32)	-0.11 (-1.54 – 0.78)
– WDT	-0.81 (-1.75 – 1.29)	-0.87 (-1.52 – 0.50)
– TSL	-0.43 (-1.65 – 1.32)	-0.33 (-1.3 – 0.60)
– PHS	-0.67 (-2.98 – 0.48)	-1.83 (-2.98 – 0.48)
– CPT	-0.49 (-0.56 – 3.24)	0.88 (-0.56 – 2.88)
– HPT	-0.41 (-0.97 – 1.64)	-0.24 (-0.97 – 2.92)
– MDT	-0.47 (-2.47 – 1.26)	0.38 (-1.18 – 1.26)*
– MPT	-0.57 (-2.07 – 1.09)	-0.33 (-1.91 – 1.09)
– MPS	0.47 (-1.46 – 2.61)	0.58 (-0.82 – 2.46)
– VDT	0.18 (-2.27 – 1.14)	0.26 (-2.55 – 1.14)
– PPT	0.48 (-2.16 – 2.61)	0.13 (-0.71 – 1.49)
Pleasant touch	8 (0 – 10)	7 (-5 – 10)

Data are given as median with range in brackets. *p<0.05.

Abbreviations: ADS = Allgemeine Depressionsskala; CCM = corneal confocal microscopy; FIQ = Fibromyalgia Impact Questionnaire; CDT = cold detection threshold; CPT = cold pain threshold; GCPS = Graded Chronic Pain Scale; HPT = heat pain threshold; IENFD = intraepidermal nerve fiber density; MDT = mechanical detection threshold; MPS = mechanical pain sensitivity; MPT= mechanical pain threshold; NBD = nerve branching density; NFD = nerve fiber density; NFL = nerve fiber length; NPSI = Neuropathic Pain Symptom Inventory; PCS = Pain Catastrophizing Scale; PHS = paradoxical heat sensation; PPT = pressure pain threshold; QST = Quantitative Sensory Testing; SSS = symptom severity score; STAI = State-Trait Anxiety Inventory; TSL = thermal sensory limen; VDT = vibration detection threshold; WDT = warm detection threshold; WPI = widespread pain index.

Supplementary Table 2. Laboratory normative values for intraepidermal nerve fiber density (normative values are not age-adjusted).

Distal IENFD (fibers/mm)	9 $^{+}$/- 3
Proximal IENFD (fibers/mm)	12 $^{+}$/- 4

Data are given as median with range in brackets.

Abbreviations: IENFD = intraepidermal nerve fiber density.

Supplementary Table 3. Correlation of cutaneous and corneal innervation with age, BMI, and pain duration in men with FMS and male healthy controls.

**FMS**			
	**Age**	**BMI**	**Pain duration**
IENFD distal (n = 42)	-0.045, p = 0.779	-0.217, p = 0.168	-0.172, p = 0.275
IENFD proximal (n = 42)	-0.042, p = 0.790	-0.125, p = 0.429	-0.179, p = 0.256
NFD (n = 41)	-0,108, p = 0.501	-0.133, p = 0.409	0.004, p = 0.979
NBD (n = 41)	-0.173, p = 0.278	0.196, p = 0.219	-0.228, p = 0.151
NFL (n = 41)	-0.227, p = 0.153	-0.163, p = 0.308	-0.162, p = 0.312
**Healthy controls**			
	**Age**		
IENFD distal (n = 55)	-0.244, p = 0.073		
IENFD proximal (n = 40)	-0.233, p = 0.148		
NFD (n = 28)	-0.276, p = 0.155		
NBD (n = 28)	-0.086, p = 0.664		
NFL (n = 28)	-0.130, p = 0.511		

The bivariate Spearman correlation was performed for correlation analysis.

Abbreviations: BMI = Body mass index; FMS = fibromyalgia syndrome; IENFD = intraepidermal nerve fiber density; NBD = nerve branch density; NFD = nerve fiber density; NFL = nerve fiber length.

Supplementary Table 4. Results of questionnaire assessment and small fiber tests in the FMS subgroups classified according to the skin innervation pattern.

	**Normal IENFD (n = 7)**	**Proximally reduced IENFD (n = 2)**	**Distally reduced IENFD (n = 11)**	**Generalized reduction of IENFD (n = 22)**	**Pathological IENFD (n=35)**
NPSI sum score<br>NPSI discriminative score	3.3 (0 – 6.4)<br>59.5 (42.0 – 71.3)	5.4 (2.7 – 8.0)<br>74.6 (74.4 – 74.7)	3.7 (1.1 – 5.9)<br>62.7 (44.8 – 77.3)	3.7 (1.6 – 7.4)<br>66.5 (44.9 – 84.3)	3.7 (1.1 – 8.0)<br>66.5 (44.8 – 84.3)
GCPS<br>– Pain intensity<br>– Disability due to pain	 57 (42 – 93)<br>40 (7 – 83)	 72 (60 – 83)<br>63 (50 – 77)	 67 (50 – 80)<br>47 (0 – 73)	 62 (37 – 90)<br>59 (2 – 83)	 63 (37 – 90)<br>53 (0 – 83)
PCS sum sore	26 (11 – 41)	4 (2 – 6)	20 (3- 38)	22 (2 – 42)	20 (2 – 42)
ADS sum sore	15 (11 – 39)	20 (7 – 33)	18 (8 – 38)	24 (8 – 48)	21 (7 – 48)
FIQ sum score	34 (28 – 58)	39 (29 – 48)	38 (27 – 67)	48 (24 – 66)	46 (24 – 67)
STAI-S<br>STAI-T	38 (37 – 63)<br>41 (36 – 59)	41 (39 – 43)<br>38 (37 – 39)	41 (32 – 73)<br>44 (33 – 73)	43 (30 – 51)<br>44 (31 – 55)	41 (30 – 73)<br>43 (31 – 73)

MPSS Gerbershagen Grad	3 (2 – 3)	3 (3 – 3)	3 (2 – 3)	3 (3 – 3)	3 (2 – 3)
WPI	10 (8 – 16)	13.5 (13 – 14)	15 (12 – 17)	13.5 (8 – 19)	14 (8 – 19)
SSS	8 (5 – 10)	6.5 (5 – 8)	7 (4 – 12)	8 (3 – 11)	8 (3 – 12)
**Small fiber tests**					
IENFD (fibers/mm)					
– distal	7.3 (6.7 – 14.4)	7.4 (6.5 – 8.3)	4.1 (1.9 – 5.9)	3.4 (1.0 – 5.9)	4.0 (1.0 – 8.3)
– proximal	12.0 (8.2 – 16.0)	3.4 (2.5 – 4.3)	9.8 (8.1 – 13.2)	6.1 (0.6 – 7.7)	6.7 (0.6 – 13.2)
CCM					
– NFD (no/mm^2)	22.9 (4.2 – 27.1)	29.2 (29.2 – 29.2)	22.9 (16.7 – 33.3)	19.3 (5.2 – 35.4)	21.4 (5.2 – 35.4)
– NBD (no/mm^2)	66.4 (28.1 – 104.2)	50.0 (50.0 – 50.0)	80.2 (21.9 – 140.8)	52.6 (13.5 – 101.6)	57.8 (13.5 – 140.8)
– NFL (mm/mm^2)	14.2 (5.0 – 15.6)	14.0 (14.0 – 14.0)	14.1 (8.4 – 18.4)	10.9 (4.8 – 17.1)	12.4 (4.8 – 18.4)
QST					
– CDT	-0.46 (-2.26 – 0.67)	0.11 (-0.59 – 0.8)	-0.04 (-1.89 – 1.32)	-0.28 (-2.26 – 1.27)	-0.26 (-2.26 – 1.32)
– WDT	-0.49 (-1.75 – 0.37)	0.35 (0-19 – 0.5)	-1.13 (-1.4 – 0.74)	-0.86 (-1.73 – 1.29)	-0.88 (-1.73 – 1.29)
– TSL	-0.17 (-1.5 – 0.6)	0.31 (0.23 – 0.38)	-0.51 (-1.65 – 0.89)	-0.43 (-1.62 – 1.32)	-0.44 (-1.65 – 1.32)

– PHS	-0.67 (-2.98 – 0.48)	-0.10 (-0.67 – 0.48)	-2.98 (-2.98 – 0.48)	-0.10 (-2.98 – 0.48)	-0.67 (-2.98 – 0.48)
– CPT	0.23 (-0.56 – 2.96)	0.69 (-0.56 – 1.94)	-0.41 (-0.56 – 2.98)	-0.14 (-0.56 – 3.24)	-0.41 (-0.56 – 3.24)
– HPT	0.26 (-0.88 – 1.64)	0.98 (-0.97 – 2.92)	-0.43 (-0.97 – 0.73)	-0.45 (-0.97 – 1.39)	-0.43 (-0.97 – 2.92)
– MDT	0.1 (-1.65 – 0.77)	0.64 (0.05 – 1.22)	-0.47 (-1.54 – 1.26)	-0.44 (-2.47 – 1.26)	-0.26 (-2.47 – 1.26)
– MPT	-0.8 (-1.96 – -0.33)	1.04 (0.99 – 1.09)	-0.8 (-1.96 – 1.09)	-0.41 (-2.07 – 0.83)	-0.33 (-2.07 – 1.09)
– MPS	0.57 (-1.46 – 1.65)	0.73 (-1.01 – 2.46)	0.58 (-0.77 – 1.66)	0.35 (-0.82 – 2.61)	0.48 (-1.01 – 2.61)
– VDT	0.18 (-1.05 – 1.14)	-0.82 (-2.55 – 0.91)	0.01 (-1.7 – 1.14)	0.36 (-2.27 – 1.14)	0.18 (-2.55 – 1.14)
– PPT	0.71 (0.03 – 1.94)	-0.24 (-1.97 – 1.49)	1.03 (-2.16 – 2.0)	0.29 (-0.83 – 2.61)	0.42 (-2.16 – 2.61)
Pleasant touch	8 (0 – 10)	1.5 (-5 – 8)	8 (0 – 10)	10 (0 – 10)	8 (-5 – 10)

Data are given as median with range in brackets.

Abbreviations: ADS = Allgemeine Depressionsskala; CCM = corneal confocal microscopy; FIQ = Fibromyalgia Impact Questionnaire; CDT = cold detection threshold; CPT = cold pain threshold; GCPS = Graded Chronic Pain Scale; HPT = heat pain threshold; IENFD = intraepidermal nerve fiber density; MDT = mechanical detection threshold; MPS = mechanical pain sensitivity; MPT= mechanical pain threshold; NBD = nerve branching density; NFD = nerve fiber density; NFL = nerve fiber length; NPSI = Neuropathic Pain Symptom Inventory; PCS = Pain Catastrophizing Scale; PHS = paradoxical heat sensation; PPT = pressure pain threshold; QST = Quantitative Sensory Testing; SSS = symptom

severity score; STAI = State-Trait Anxiety Inventory; TSL = thermal sensory limen; VDT = vibration detection threshold; WDT = warm detection threshold; WPI = widespread pain index.
